# Acute Kidney Injury in Diabetes Mellitus

**DOI:** 10.1155/2016/6232909

**Published:** 2016-11-15

**Authors:** D. Patschan, G. A. Müller

**Affiliations:** Clinic of Nephrology and Rheumatology, University Hospital of Göttingen, Göttingen, Germany

## Abstract

Diabetes mellitus (DM) significantly increases the overall morbidity and mortality, particularly by elevating the cardiovascular risk. The kidneys are severely affected as well, partly as a result of intrarenal athero- and arteriosclerosis but also due to noninflammatory glomerular damage (diabetic nephropathy). DM is the most frequent cause of end-stage renal disease in our society. Acute kidney injury (AKI) remains a clinical and prognostic problem of fundamental importance since incidences have been increased in recent years while mortality has not substantially been improved. As a matter of fact, not many studies particularly addressed the topic “AKI in diabetes mellitus.” Aim of this article is to summarize AKI epidemiology and outcomes in DM and current recommendations on blood glucose control in the intensive care unit with regard to the risk for acquiring AKI, and finally several aspects related to postischemic microvasculopathy in AKI of diabetic patients shall be discussed. We intend to deal with this relevant topic, last but not least with regard to increasing incidences and prevalences of both disorders, AKI and DM.

## 1. Introduction and Aim

The incidence and prevalence of diabetes mellitus (DM) have continuously been increased over the last 20 years. Meanwhile an estimated number of 387 million people worldwide suffer from DM [1]. Morbidity and mortality of diabetic patients are substantially aggravated by cardiovascular complications including coronary artery, cerebrovascular, and peripheral artery disease. In addition, DM may significantly affect kidneys and urinary tract. The disease accounts for most cases of end-stage renal disease in Western-Europe and in the US. Approximately 40% of all patients requiring dialysis therapy on a regular basis suffer from diabetes mellitus as respective cause [2]. Chronic renal insufficiency results from both, extra- and intrarenal atherosclerosis, and from diabetes-associated glomerular damage (diabetic nephropathy). In addition, diabetic kidneys are characterized by severe interstitial inflammation [3]. Finally, patients are at higher risk for developing contrast-induced nephropathy (CIN) [4] and frequently suffer from bacterial infections, often involving urinary tract and renal tissue* per se*.

Acute kidney injury (AKI) on the other hand remains a fundamental problem in hospitalized patients worldwide. As a matter of fact, incidences have also increased steadily in recent years, reaching almost 20% in middle-Europe. This most likely results from ageing of the population in general, accompanied by an overall increased morbidity [5]. A meta-analysis from 2013 that evaluated more than 300 studies showed an average AKI world-incidence of even more than 30% in adults [6]. Nevertheless, mortality rates have only mildly been improved since the early 1990s. Zeng and colleagues reported dramatical differences in AKI mortality, depending on the severity of the syndrome [7]. If related to the AKIN criteria [8] overall in-hospital mortality rates were as follows: no AKI 0.6%, AKIN stage I 5.3%, AKIN stage II 13.4%, and AKIN stage III 35.4%. The lowest survival rates have been reported in patients suffering from a malignant disorder and from sepsis-associated AKI with the need for renal replacement therapy. In this particular group, mortality can exceed even 90% [9].

Regarding not only the individual but also the epidemiological and economical consequences of both disorders, DM and AKI, it is somehow surprising how few studies addressed the topic “AKI in diabetes mellitus.” This article is intended to summarize the current knowledge of three aspects related to the field. Firstly, we will give an epidemiological overview: how does diabetes affect AKI risk and survival? Do diabetic patients suffer from an overall higher morbidity after AKI? Secondly, we will discuss the importance of blood glucose control at the intensive care unit (ICU) with special attention to AKI incidences and survival. Finally, several experimental investigations will be discussed. We will especially focus on microvascular or endothelial dysfunction which may emerge early during the course of the disease, thus potentially increasing ischemia-vulnerability of the organ.

## 2. AKI Epidemiology in Diabetes Mellitus

Several studies evaluated AKI epidemiology in diabetic patients. Mehta and colleagues [10] performed a retrospective analysis, based on the* Society of Thoracic Surgeons National Database. *All patients included between 2002 and 2004 were analyzed, with a total number of 449,524 individuals. The total prevalence of DM was 33%. Dialysis treatment became mandatory in 6,451 patients after surgery. In individuals requiring dialysis, diabetes was diagnosed more frequently than in those without renal replacement therapy (49 versus 33%, *p* < 0.0001). In addition, more detailed analysis using a multivariate logistic regression model revealed diabetes as independent risk factor for developing AKI after cardiac surgery. Another study published by Oliveira and colleagues [11] prospectively evaluated patients undergoing aminoglycoside treatment (*n* = 980). The primary endpoint was a reduction in the glomerular filtration rate (GFR) of 20% or more. The diabetes prevalence was 19.6% in patients that fulfilled the endpoint versus 9.3% without GFR reduction (*p* = 0.007). Comparable to the study by Mehta et al. [10] Oliveira and colleagues performed logistic regression analyses as well. These showed several independent AKI risk factors: baseline GFR of <60 mL/min/1.73 m^2^, the use of iodinated contrast media, hypotension, concomitant use of nephrotoxic drugs, and diabetes (OR, 2.13; 95% CI, 1.01 to 4.49; *p* = 0.046). Girman et al. [12] retrospectively performed a survey of the* General Practice Research Database* (UK), comparing 119,966 type 2 DM patients with 1,794,516 nondiabetic individuals. The yearly AKI incidence was 198 versus 27/100,000 subjects, and the difference remained statistically significant even after adjustment for other well-known AKI risk factors and comorbidities. At this point it needs however to be mentioned that diabetic patients displayed an overall higher cumulative morbidity in general. They differed in the following categories: obesity, congestive heart failure, hypertension, alcohol and tobacco exposure, past AKI episodes, CKD prevalence, therapy with ACE inhibitors/angiotensin receptor blockers, therapy with other antihypertensive drugs, statin treatment, and NSAID use (*p* values in every category below 0.001). Hsu and colleagues compared 1,746 hospitalized adults (*Kaiser Permanente Northern California*) that developed dialysis-requiring AKI with over 600,000 individuals without such a complication [13]. The following parameters were identified as independent AKI risk factors: preadmission diabetes mellitus, arterial hypertension, and preexisting proteinuria.

The study by Thakar, performed in a prospective manner, somehow differed from the other investigations since it exclusively evaluated type 2 diabetic patients (*VA healthcare system*). The primary endpoint was progression towards CKD stage 4, depending on several risk factors and in particular depending on the presence of AKI during the observational period (01/1999–12/2004). General risk factors for CKD progression were arterial hypertension, obesity, and higher average age. Kidney-related risk factors were initial proteinuria, a lower mean GFR at the beginning of the study, and AKI* per se*. It also became apparent that survival probability gradually decreased with increasing number of AKI episodes. Finally, mortality increased further with lower initial mean GFR. Recently, Venot et al. [14] published an investigation designed as prospective case-control study. Three-hundred and eighteen diabetic patients were compared with 746 nondiabetic controls, and patients in both groups suffered from either severe sepsis or from septic shock. Interestingly, AKI incidences did not differ between the two groups but surviving subjects with diabetes more often required dialysis at discharge, showed higher mean serum creatinine levels, and did recover less efficiently than nondiabetics. The study by Venot et al. has several limitations which were discussed in the original manuscript in detail. First, the diagnosis of diabetes was made according to the medical history available from patients/relatives/consultants, HBA1C levels were not incorporated neither were diabetic long-term complications. Next, preexisting CKD was not defined as exclusion criterion since in some patients exact information about the initial status of kidney function was missing. Third, the diagnosis of AKI was made according to the KDIGO criteria [15] but without including urine output rates which were not documented. Finally, the need for dialysis treatment was not determined according to standardized criteria but was evaluated by clinicians from different sites in individual manners. Together, these confounding factors may potentially account for the lack of differences between diabetic and nondiabetic patients.

Two additional investigations confirmed these findings [16, 17].  Table 1 summarizes the results of the mentioned studies related to the topic. In summary the current data, although not consistently acquired in a controlled and prospective manner, indicate a higher AKI risk in the presence of (type 2) DM and they suggest a higher AKI-morbidity and mortality risk if patients suffer from the diabetic disease. An additional remark may be allowed with regard to all of the studies discussed above: they (almost) all investigated patients treated in the intensive care unit. By far not every patient who develops AKI during in-hospital treatment is being transferred to the intensive care unit. Therefore, a vast amount of outcome data may be missing in these investigations.

Another aspect that needs to be discussed is AKI risk in relation to preexisting diabetic nephropathy and to other coexisting morbidities. The currently available data suggest a higher AKI risk in diabetic individuals as compared to nondiabetic persons but it remains unclear whether this association is attributable to the hyperglycemic milieu* per se* or if it potentially results from end-organ damage such as generalized and intrarenal atherosclerosis. Only very few studies evaluated this particular aspect. Vallon [18] brought up the question whether changes in tubular homeostasis in diabetic nephropathy may increase AKI risk or not. In the end, any mechanistic relationship between diabetes-induced upregulation of TGF-*β*, (premature) senescence, and inflammation could only be suggested.

To conclude the whole section, it has to be realized that AKI risk is most likely being increased in diabetic individuals. Nevertheless, the pathophysiological determinants responsible for such association are currently unknown. It needs to be elucidated more in detail how diabetic and nondiabetic comorbidities potentially increase the risk for acute kidney injury in diabetes mellitus.

## 3. Blood Glucose Control at the Intensive Care Unit: Impact on AKI Incidence and Survival

Chronic hyperglycemia is a well-known risk factor for atherosclerosis. Elevated blood glucose levels have nevertheless also been associated with impaired outcomes in acute situations such as myocardial infarction and stroke [19–21]. The mechanisms responsible may include hyperglycemia-induced release of free fat acids, the inactivation of nitric oxide (NO), and increased production of reactive oxygen species (ROS) [19], respectively. A number of studies compared glucose control in the intensive care unit by either conventional or intensified (continuously administered) insulin therapy with regard to AKI incidences. Thomas et al. reviewed the results in a 2007 published meta-analysis [22]. Three controlled, randomized, and prospective and 2 noncontrolled, prospective studies were included [23–27]. In all included studies, the AKI risk-ratio (RR) was below 1 in patients undergoing intensified insulin therapy (0.74 [24], 0.66 [23], 0.15 [27], 0.25 [26], and 0.67 [25]). Nevertheless, in two of the above mentioned (randomized controlled) studies [23, 25] the 95% confidence intervals were up to 0.99, respectively. Thus, differences between conventional and intensified insulin treatment regimens were only mild. One study in contrast showed significant AKI protection under intensified glucose control [27]. However, it may in addition not be concluded that stricter protocols for glucose control exclusively improve outcomes of ICU patients. The NICE-SUGAR [28] trial compared ICU patients receiving glucose control in a strict (81–108 mg/dL) versus a liberal (>108 and below 180 mg/dL) manner. Three thousand fifty-four patients were assigned to the first and 3,050 individuals to the second group in a prospective manner. As a matter of fact, AKI incidences did not differ between the two categories but survival was significantly lower in those receiving a stricter insulin therapy (*n* = 829—27.5% versus 751—24.9% with *p* = 0.02). It needs to be noted that severe hypoglycemia, defined as blood glucose levels of below 40 mg/dL, occurred in 206 (6.8%) patients in the first versus 15 (0.5%) patients in the second group (*p* < 0.001). The 2012 published version of the “KDIGO clinical practice guidelines for acute kidney injury” [15] therefore recommended target glucose levels of 110–149 mg/dL to be achieved in ICU patients. Thus, AKI incidences may be reduced without further aggravating mortality.

## 4. Microvascular Dysfunction and AKI in Diabetes Mellitus

Hyperglycemia is a well-known risk factor for endothelial dysfunction [29]. Even quite early after being exposed to a hyperglycemic milieu, for instance, induced by the administration of “advanced glycation end-products” (AGEs), cultured endothelial cells show impaired production of nitric oxide which reflects the loss of cellular competence [29]. In addition, hyperglycemia has also been proven as inductor of premature endothelial senescence (stress-induced premature senescence—SIPS) [30]. The term “senescence” describes the process of functional and structural ageing of cells. Its first description was made by Hayflick and Moorhead who observed inhibition of fibroblast proliferation during cell culturing for several weeks [31]. Such “replicative type” of cellular senescence must be differentiated from another process of ageing that results from pathological stimuli such as oxidative stress [32], poor cell culture conditions [30], and/or the activation of certain (proto)oncogenes [33]. This second type has been defined as “stress-induced premature senescence” or SIPS [34]. According to current concepts, SIPS results from intracellular accumulation of telomeres, ultimately leading to DNA damage [35]. At the end of this section we will address potential pathophysiological consequences of DM-induced endothelial SIPS in AKI.

The importance of microvascular dysfunction in AKI has been highlighted by numerous experimental investigations. In most cases AKI ensues from transient renal hypoperfusion or ischemia [5]. The hallmark in ischemic AKI (iAKI) is tubular cell dysfunction and damage. The respective cellular and molecular mechanisms have extensively been studied and reviewed in the past [29–32]. In addition, ischemia also induces significant interstitial inflammation and functional impairment/structural damage of small peritubular and glomerular blood vessels [29, 30, 33–35]. The inflammatory response is being initiated by both tubular and vascular malfunction and encompasses the activation of virtually all components of the innate and acquired immune system [30]. The topic “postischemic inflammation” should meanwhile be recognized as a separate area in the field [35]. Microvascular damage on the other hand significantly affects the kidney in the short- and the long-term. Short-term effects involve endothelial cell expansion and apoptosis/necrosis, both resulting in microvascular obstruction. Thus, postischemic reperfusion is inhibited and kidney regeneration is prolonged [36, 37]. In addition, every ischemic insult diminishes the intrarenal total vascular surface area, subsequently followed/accompanied by endothelial-to-mesenchymal transdifferentiation (EndoMT) [38–40]. The ultimate consequence is aggravated fibrosis and an increased risk for CKD [33, 41, 42]. Nevertheless, the impact of diabetes-induced endothelial dysfunction (ED) on ischemia-vulnerability of the kidney has only sporadically been investigated in the past. Goor and colleagues [43] analyzed a model of type I DM. Rats were repeatedly injected with streptozotocin, followed by oral supplementation with the substance N-omega-nitro-L-arginine, a well-known nitric oxide synthase inhibitor. Fourteen days later, animals were subjected to ischemia-reperfusion injury. Postischemic creatinine clearance was significantly higher in nondiabetic animals (163 ± 30 versus 90 ± 22 *μ*L/min/100 g; *p* < 0.005). In addition, only nondiabetic rats showed increased serum and urinary levels of nitric oxide (NO) despite pharmacological NO synthesis inhibition. Finally, animals from both groups were provided with the NO donor L-arginine. This measure exclusively improved kidney function in nondiabetic rats. Taken together, this study has several important implications. Firstly, nondiabetic animals were capable of producing NO even after exogenous NO synthesis inhibition, most likely by alternative mechanisms. Secondly, nondiabetic animals still showed sensitivity towards NO which predominantly acts on the microvascular level. In other words, quite early after diabetes induction (14 days), animals displayed severe endothelial dysfunction (reduced NO synthesis and diminished NO sensitivity) resulting in aggravated postischemic kidney damage. Another study of interest was published by Shi and colleagues in 2007 [44]. By using Laser-Doppler Flowmetry, postischemic kidney reperfusion was analyzed in both compartments, the renal medulla and the cortex. Two parameters were of interest: postischemic flow acceleration and time until complete normalization of capillary flow. In contrast to the study by Goor et al. [43] diabetes was not induced by pharmacological measures but the authors used a well-established genetic model of type II diabetes, db/db mice [45]. These animals suffer from severe insulin resistance and obesity. As a matter of fact, both outcome parameters were significantly impaired in db/db as opposed to db/m and to wildtype control mice: postischemic flow acceleration was reduced and time until flow normalization was significantly longer in both compartments. It has to be noted that animals were only 10–12 weeks old. In summary, this study also points towards microvascular or endothelial dysfunction to occur quite* early* during the diabetic disease and it indicates that DM-induced ED alone may significantly affect ischemia-vulnerability of the kidney.

Another study also revealed diabetes as “fast-acting” risk factor for AKI, although it did not focus on diabetes-associated endothelial dysfunction. Rats were subjected to renal ischemia at week 2 after finishing a diabetes induction protocol using the substance streptozotocin [46]. Animals were analyzed 4 and 8 weeks later. Nondiabetic rats almost completely recovered from functional impairment and tissue damage while diabetic rats showed extensive inflammation and tubulointerstitial fibrosis at week 4. At week 8, diabetic kidneys were even reduced in mass, resulting from severe tubular loss. It may however not be forgotten that increased ischemia-sensitivity of the renal tissue in diabetes does exclusively result not only from impaired vascular (endothelial) function but also from aggravated inflammation. This particular topic shall however not be discussed at the moment. We would rather like to refer to some excellent manuscripts on the subject [35, 47, 48].

Finally, we would like to briefly mention a study recently published by Peng and colleagues [49]. Streptozotocin- (STZ-) treated mice showed higher ischemia-vulnerability than nondiabetic controls, although ischemia was applied quite early (3 weeks) after finishing the STZ protocol. Even though this model cannot truly be defined as model of diabetic nephropathy which usually evolves years after onset of diabetes in humans, it became apparent that hyperglycemia substantially induced p53 both,* in vivo* and* in vitro*. Subsequently, mitochondrial release of cytochrome C was increased as well. The ischemic damage decreased upon p53 silencing using siRNA. Together, the study offered a new mechanistic perspective on processes responsible for increased ischemia-sensitivity of the kidney in diabetes [50].

Finally, we would like to discuss possible implications of DM-induced endothelial SIPS in AKI. Although it must be understood as a rather pathological event, the hallmark of SIPS is faster or premature ageing of cells. The functional consequences more or less correspond to those that occur during normal ageing of tissues/organs. As pointed out earlier, AKI incidences have steadily increased in recent years, significantly resulting from ageing of the population in general [5]. Higher age-related susceptibility of the kidney has been reported by several investigators [49, 50]. Schmitt and Cantley [51] reviewed the mechanisms that may increase kidney vulnerability in older individuals in an excellent manner [50]. Numerous processes were discussed including declined capacity of renal epithelial cells to proliferate, impaired function of certain types of stem and progenitor cells, and alterations of renal growth factor profiles. Clements and colleagues demonstrated exacerbation of vascular rarefication and CKD risk in aged mice following ischemia-reperfusion injury [52]. Nevertheless, the investigation was not performed under diabetic circumstances. A dynamic cascade of hyperglycemia-associated endothelial SIPS, accompanied by reduced autophagy, has been reported by Goligorsky and colleagues [53]. Autophagy (AP) is widely regarded as endogenous mechanism of self-protection/repair [54]. Own studies showed that TGF-beta-induced SIPS of cultured early endothelial progenitor cells goes in parallel with diminished AP as well [55]. As a matter of fact, intrarenal endothelial autophagy stimulation by pharmacological measures reduced mesenchymal transition of endothelial cells (EndoMT) after AKI [55]. EndoMT has been repeatedly reported to aggravate kidney fibrosis [38, 39]. One may therefore argue that endothelial SIPS, induced either by ischemia* per se* or by prolonged hyperglycemia, promotes EndoMT and fibrosis while increased AP mediates the opposite. Recently, we reported increased EndoMT and endothelial SIPS to occur in diabetic nephropathy. However, no investigation published so far analyzed endothelial SIPS in AKI under diabetic circumstances. We therefore recently initiated a project related to this particular topic. We intend to reduce endothelial SIPS by pharmacological AP stimulation after AKI. We sincerely hope to identify stress-induced cellular senescence as hallmark of aggravated postischemic endothelial damage within the diabetic microenvironment. Thus, antisenescent therapeutic strategies should be tested and hopefully established in order to improve microvasculopathy and AKI outcomes in the short- and long-term.

**Figure 1:**
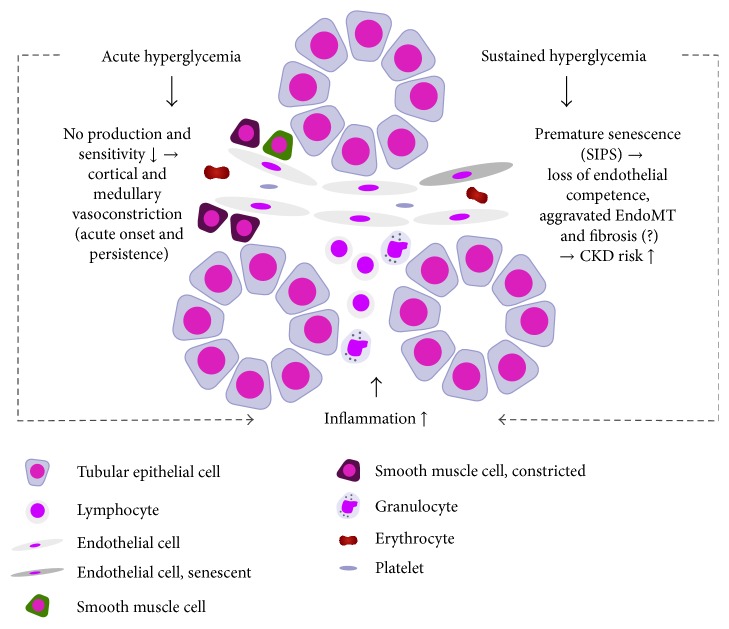
Pathophysiological consequences of DM on postischemic MV in AKI.

Figure 1 summarizes pathophysiological aspects of DM-associated microvasculopathy in AKI.

## 5. Conclusions

- In summary, we conclude that DM potentially increases AKI risk and long-term mortality/morbidity of AKI.
- In the intensive care unit, blood glucose levels should be adjusted to high-normal/mildly increased levels. Thus, AKI risk may be minimized without elevating mortality rates.
- Finally, DM should be recognized as “fast-acting” risk factor for kidney vulnerability to ischemia. The tissue susceptibility increases as a result of significant microvasculopathy and of interstitial inflammation. The latter effects can occur even in nondiabetic patients in whom acute blood glucose deterioration is not efficiently controlled.

## Figures and Tables

**Table 1:** Selected studies that evaluated AKI incidences and outcomes in diabetic patients. For detailed description see text.

Study/year	Design	Results
Mehta et al., 2006 [10]	Retrospective, data-based analysis (*Society of Thoracic Surgeons National Database*), DM prevalence in AKI patients after cardiac surgery; included individuals: 449,524	DM prevalence 49 versus 33% in AKI versus no AKI (*p* < 0.0001)
Oliveira et al., 2009 [11]	Prospective single-center analysis, DM prevalence in aminoglycoside-induced AKI; included individuals: 980	DM prevalence 19.6 versus 9.3% in AKI versus no AKI (*p* = 0.007)
Girman et al., 2012 [12]	Retrospective, data-based analysis (*General Practice Research Database*), AKI in DM versus no DM; included individuals: 119,966 type 2 DM patients and 1,794,516 nondiabetic individuals	Yearly AKI incidence in DM versus no DM: 198 versus 27/100,000 subjects
Venot et al., 2015 [14]	Prospective case-control study, AKI incidences and outcomes of patients with severe sepsis/septic shock, DM versus no DM; included individuals: 318 diabetic and 746 nondiabetic controls	AKI incidences not different but dialysis frequency and serum creatinine at discharge higher in DM
Kheterpal et al., 2009 [16]	Retrospective, data-based analysis (American College of *Surgeons National Surgical Quality Improvement Program*), AKI incidence after general surgery; included individuals: 75,952	Identification of DM as independent preoperative risk factor
Mittalhenkle et al., 2008 [17]	Prospective case-control study (*Cardiovascular Health Study*), AKI incidence in the elderly; included individuals: 5,731	Association of DM with incident acute renal failure (AKI)
